# Visual acuity in various phenotypes of intermediate age related macular degeneration (AMD) in a multicentre cohort study in Europe- INTERCEPT-AMD report 1

**DOI:** 10.1038/s41433-025-03895-y

**Published:** 2025-07-19

**Authors:** Sarega Gurudas, Inês Marques, Jean-François Girmens, Yara Lechanteur, Maria Cristina Parravano, Lieselotte Berger, Hansjürgen Agostini, Sandra Barrão, Evangelos Tsiroukis, Jordi Monés, Laura Sararols, Rufino Silva, Hendrik Scholl, Albrecht Lommatzsch, Boris Stanzel, Stela Vujosevic, Sobha Sivaprasad, On behalf of the INTERCEPT-AMD Study Group, Inês Marques, Jean-François Girmens, Yara Lechanteur, Maria Cristina Parravano, Lieselotte Berger, Hansjürgen Agostini, Sandra Barrão, Evangelos Tsiroukis, Jordi Monés, Laura Sararols, Rufino Silva, Albrecht Lommatzsch, Boris Stanzel, Stela Vujosevic, Sobha Sivaprasad, Hendrik P. N. Scholl, Yannick Liermann, Paolo Lanzetta, Emily Fletcher, Savita Madhusudhan, Lyubomyr Lytvynchuk, Francesco Bandello, Nicole Eter, Stefano de Cilla, Michel Weber, Aude Ambresin

**Affiliations:** 1 National Institute of Health Research Moorfields Biomedical Research Centre, Moorfields Eye Hospital, London, UK; 2 Centre for Clinical Trials, AIBILI / Association for Innovation and Biomedical Research on Light and Image, Coimbra, Portugal; 3 Centre d’Investigation Clinique, Centre National d’Ophtalmologie des Quinze-Vingts, Paris, France; 4 Department of Ophthalmology, Radboud University Medical Centre, Nijmegen, Netherlands; 5 IRCCS Fondazione G.B. Bietti per lo Studio e la Ricerca in Oftalmologia ONLUS, Rome, Italy; 6 Department of Ophthalmology, Inselspital, University of Bern, Bern, Switzerland; 7 Department of Ophthalmology, University of Freiburg, Freiburg, Germany; 8 Instituto de Oftalmologia Dr. Gama Pinto, Lisboa, Portugal; 9 Institut Català de Retina (ICR), Clinical Trial Unit, Barcelona, Spain; 10 Institut de la Màcula, Centro Médico Teknon, Barcelona, Spain; 11 Valles Ophthalmology Research, S.L. Barcelona, Spain; 12 Espaço Médico de Coimbra, Coimbra, Portugal; 13 University Hospital Basel, University Eye Clinic, Basel, Switzerland; 14 Department of Ophthalmology, St. Franziskus-Hospital, Münster, Germany; 15 Eye Clinic Sulzbach, Knappschaft Hospital Saar, Sulzbach, Germany; 16 Medical Retina Service, Operative Unit Ophthalmology—MultiMedica Spa (IRCCSMM), Milan, Italy; 17 NIHR Moorfields Clinical Research Facility, Moorfields Eye Hospital, NHS Foundation Trust, London, UK; 18 Department of Ophthalmology University of Bonn, Bonn, Germany; 19 Department of Ophthalmology, University of Udine, Udine, Italy; 20 Clinical Trial Unit, Dep. Ophth., Gloucestershire Hospitals NHS Foundation Trust, Cheltenham, UK; 21 Clinical Eye Research Centre—St. Paul’s Eye Unit, Royal Liverpool University Hospital, Liverpool, UK; 22 Department of Ophthalmology, Justus-Liebig-University-Giessen, Giessen, Germany; 23 Department of Ophthalmology, University Vita Salute—Scientific Institute of San Raffaele, Milan, Italy; 24 Department of Ophthalmology, University of Muenster Medical Center, Münster, Germany; 25 Eye Unit, University Hospital Maggiore della Carità, Novara, Italy; 26 Department of Ophthalmology, University Hospital, Nantes, France; 27 Swiss Visio Retina Research Center, Swiss Visio Montchoisi, Lausanne, Switzerland

**Keywords:** Biomarkers, Prognosis, Epidemiology

## Abstract

**Background/Objectives:**

To study the associations of VA and intermediate age-related macular degeneration (iAMD) subclassifications.

**Subjects/Methods:**

This is the analysis of baseline data of a multicentre study on patients with iAMD in at least one eye. The subclassifications of iAMD were classified as: (i) iAMD with no evidence of incomplete retinal pigment epithelial and outer retinal atrophy (iRORA) or subretinal drusenoid deposits (SDD); (ii) iAMD with SDD with no iRORA; (iii) iAMD with iRORA with no SDD and (iv) iAMD with iRORA and SDD.

**Results:**

983 eyes from 805 patients with iAMD were analysed. The mean age was 75.8 years (SD 7.9), with 35.0% (282) male. Eyes with iRORA with SDD had lower VA relative to eyes with no iRORA and no SDD (OR = 0.98 [95% CI 0.96, 0.998]; *P* = 0.03). The VA in the better seeing eye was significantly higher than in the worse seeing eye. Increased age (sex adjusted OR, 1.07, 95% CI 1.05–1.09; *P* < 0.001) and female gender (age adjusted OR, 0.75, 95% CI 0.56–1.01; *P* = 0.057) were associated with SDD. Eyes with nAMD in the fellow eye had reduced odds of iRORA in the study eye (adjusted OR = 0.60, 95% CI 0.40–0.91; *P* = 0.015). Eyes with GA had increased odds of iRORA in the study eye (adjusted OR = 3.30, 95% CI 2.00–5.45; *P* < 0.001).

**Conclusions:**

Baseline age, presence of SDD and/or iRORA and fellow eye status need to be considered in future clinical trials evaluating preventive or treatment options for iAMD.

## Introduction

Advanced age-related macular degeneration (AMD) is the commonest cause of irreversible central vision loss in the elderly [1]. A susceptible eye progresses through various severity levels before development of advanced AMD [2, 3]. Most patients only notice visual impairment when the disease has progressed to the advanced forms of either geographic atrophy (GA) or neovascular AMD (nAMD) and these forms may co-exist [4, 5]. Therefore, visual acuity (VA) is not a sensitive functional measure until the advanced stages of disease [4]. However, regulatory agencies require interventions for AMD to achieve an endpoint of at least 5-letter gain in eyes with baseline VA of 20/100 Snellen or better. A responder definition is a ≥ 15-letter change relative to baseline [6, 7]. This may be a prevention of 15 or more letters lost or a 15-letter gain in VA. Therefore, baseline VA is a strong determinant of final visual outcome, an important consequence of transition to both advanced forms [8].

Currently, there are significant variations in mean baseline VA in clinical trials evaluating preventive options for progression to advanced AMD. For example, the baseline median VA of the participants of the LEAD trial on subthreshold laser for iAMD was 83 (IQR 80–89) letters in the treatment arm and 84 (1QR 79–88) letters in the sham arm [9]. In contrast, the LIGHTSITE III trial on iAMD had a baseline mean VA of 70.7 (SD 5.23) letters in the intervention arm and 70.1 (SD 4.29) letters in the sham arm [10]. Although these studies had different inclusion criteria for VA, these differences highlight the need for further stratification of trial participants to ensure that homogeneous cohorts are compared in trial arms and between trials.

AMD is classified into early, intermediate, and advanced stages based on colour fundus photography [2, 3]. Eyes with intermediate AMD (iAMD) have a high risk of progression to advanced AMD and are therefore the chosen cohort for AMD prevention trials. On multimodal imaging, especially optical coherence tomography (OCT), several high-risk features in eyes with iAMD have been identified that contribute to disease progression. Amongst them include the presence of subretinal drusenoid deposits (SDD) and incomplete retinal and retinal pigment epithelial atrophy (iRORA) that are both risk factors for GA [11–14]. On average, it only takes seven months for iRORA to transition to complete retinal and retinal pigment epithelial atrophy or cRORA [14, 15]. Therefore, categorising iAMD based on the presence or absence of these features may help to inform future trial designs [16, 17].

Moreover, inclusion criteria for iAMD may include either one eye of bilateral iAMD, which may be the better or worse seeing eye, or the second eye with iAMD in patients with unilateral advanced AMD. The VA differences at baseline between these categories may significantly impact the choice of study eye for clinical trials on iAMD.

This is a collaborative multicentre study within EVICR.net Members in Europe to evaluate course of iAMD by two-years.

Key objectives of this baseline report were to: (a) assess whether there are any differences in VA between better or worse seeing eye with bilateral iAMD compared to second eye with iAMD in patients with unilateral nAMD or GA; (b) characterise iAMD into various imaging subgroups and study their relationship to VA and (c) evaluate whether there are any demographic or imaging characteristics that are associated with VA.

## Methods

### Design

This multicentre retrospective and prospective study was organised by EVICR.net Coordinating Centre, Coimbra, Portugal that obtained the overarching institutional review board approval for the study. Each site then obtained their own regulatory approval required for each country, where applicable. The study protocol was approved by AIBILI Ethics Committee for approval. Informed consent was obtained from patients from countries where it is a regulatory requirement. For countries where it was not a regulatory requirement it was because it was considered a retrospective data collection.

### Patient eligibility

Patients with iAMD in at least one eye based on the Beckman Classification^2^, and had at least 3 clinic visits with multimodal imaging across two years were identified from respective electronic medical records and imaging datasets and included in the study. Multimodal imaging included mandated Spectralis macular OCT scans with infrared imaging (IR). Fundus autofluorescence (FAF) and enhanced depth imaging of OCT were optional. Eyes with poor-quality images as determined by site investigators were excluded. Other exclusion criteria included eyes with bilateral GA or nAMD, any other co-existent ocular diseases or any patient who had opted out of their information being used for research nationally or locally at any Member Site. These criteria were screened by site personnel and reasons for exclusion as a study eye were recorded, where possible. In addition to the images, routine demographic data (age and sex) and available VA records were entered in the database. The VA was recorded in ETDRS letters at all sites. Methods of recording measurement of VA included VA with pinhole, VA with patient’s glasses or best-corrected visual acuity (BCVA).

### Selection of study eye

If both eyes met the eligibility criteria of iAMD, they were both enroled as study eyes. The eye with better VA was included if a patient-level VA was considered. If only one eye had iAMD, the macula status of non-study eye was graded on multimodal imaging as: presence of cRORA; nAMD, early AMD, healthy macula or excluded due to poor image quality or due to other co-existent macular pathology.

Each eye with iAMD was further classified by the site personnel into the following pre-defined categories: (i) iAMD with no atrophy and no SDD; (ii) iAMD with no atrophy with SDD; (iii) iAMD with iRORA with no SDD, and (iv) iAMD with iRORA with SDD [16]. The Classification of Atrophy Meetings (CAM) group was used to define iRORA and cRORA on OCT [13, 15]. These include a region of signal hypertransmission into the choroid of <250 μm, a corresponding zone of attenuation or disruption of the RPE, with or without the persistence of basal laminar deposits (BLamD) and evidence of overlying photoreceptor degeneration, i.e., subsidence of the inner nuclear layer (INL) and outer plexiform (OPL), presence of a hyporeflective wedge in the Henle fibre layer (HFL), thinning of the outer nuclear layer (ONL), disruption of the external limiting membrane (ELM), or disintegrity of the ellipsoid zone (EZ), and when these criteria do not meet the definition of complete retinal pigment epithelium and outer retinal atrophy (cRORA) that defines geographic atrophy on colour photographs. SDD was defined on at least two of three image modalities; IR, OCT or FAF.

### Data collection

The EVICR.net established data sharing agreements from sites to the EVICR.net Eye platform and only anonymised data and images were transferred to the Coordinating Investigator (Moorfields Eye Hospital, London, UK) via the EVICR.net Eye platform. The visit window allowed for a margin of ±3 months.

### Sample size

A formal sample size based on outcomes was not done as this study was aimed to collect a large research database for future studies. A sample size of 1000 eyes with VA records at baseline and over 2 years was considered a feasible resource for studying subclassification and associations with VA.

### Statistical analysis

AMD severity was compared between both eyes, including non-study eyes, to assess the level of asymmetry in the disease. Baseline demographic and ocular characteristics were summarised for each iAMD category using mean (SD) or median (IQR) for continuous variables and n (%) for categorical variables. Boxplots were used to illustrate the distribution of VA and age by iAMD severity. Baseline demographic description on the whole cohort, by VA categories and by iAMD categories in the study eye were summarised. The association between age and VA was evaluated within each iAMD category using linear mixed-effects models with random intercepts to account for within-participant correlation due to bilaterality of disease (inclusion of both eyes) in some participants. A statistical model was fitted including an interaction term between iAMD category and age to test for statistically significant differences in the association between age and VA between iAMD category. The paired t-test was used to compare VA in bilateral iAMD eyes based on best-worst seeing eye, comparing VA in eyes with nAMD in non-study eye and comparing VA in eyes with GA in the non-study eye. Intraclass correlation coefficient (ICC) obtained using the two-way mixed effects (3,1), consistency, single-measurement model, was used to assess the degree of consistency in VA between eyes from the same individual, while Spearman’s correlation coefficient to assess the strength and direction of the monotonic relationship in VA between paired eyes. A one-way Analysis of Variance (ANOVA) was used to assess age differences among patients with bilateral AMD, those with nAMD in the fellow eye, and those with GA in the fellow eye.

Logistic regression analysis was used to assess the associations between demographic variables, VA and the presence of SDD, and the presence of iRORA. Additionally, multinomial logistic regression was conducted to evaluate the associations of baseline characteristics with the fine-grained four intermediate AMD categories. Both logistic regression and multinomial logistic regression were employed using Generalized Estimating Equations (GEE) with an exchangeable correlation structure to account for within-participant correlation due to bilaterality of disease (inclusion of both eyes) in some participants. GEE models are marginal models which model a population average, therefore, the parameter estimates from a GEE model can be interpreted at the population level due to the averaging over all clusters. For the multinomial GEE model, a time-exchangeable correlation structure was assumed. Odds ratios with 95% CI and *P*-values were reported from the logistic regression models, while local Odds Ratios with 95% CI and *P*-values were reported from the multinomial logistic regression model.

VA was analysed both as a continuous variable and by grouping into the categories ≤69 ETDRS letters, 70–79 ETDRS letters, and ≥80 ETDRS letters. Age was categorized into groups <75, 75–85, and >85 years. Non-linearity was explored through categorisation and the inclusion of both linear and quadratic terms for age and VA, as well as fractional polynomials using the multivariable fractional polynomial procedure (adjusting for age and gender). The multivariable fractional polynomial (MFP) procedure identifies non-linear relationships while simultaneously performing backward elimination to select the optimal subset of predictors [18].

Complete case analysis was performed, and due to missing data in VA, the distribution of baseline characteristics was compared in eyes with complete and incomplete VA data, to ensure those that have been removed from the sample due to missing data are similar in distribution to those analysed in the main sample. Since not all eyes had their best-corrected visual acuity (BCVA) recorded, analysis was conducted to evaluate associations of BCVA using only those eyes that reported it. All statistical analyses were performed using R, with a significance level set at 5% for all tests. Data were visualized using the ggplot2 and sjPlot packages. Multinomial logistic regression and binary logistic regression via generalized estimating equations (GEE) was fit using the respective packages multgee and geepack in R. The analysis, based on unilateral eyes, used multinomial logistic regression with the VGAM package in R (IRLS algorithm) without GEE. Similarly, binary logistic regression was applied without GEE for unilateral eyes.

## Results

A total of 1638 eyes from 819 patients were evaluated. Of which, 983 eyes of 805 patients met the eligibility of iAMD and were included in the main analysis (Table S1) and 17 (1.7%) eyes had other types of macular atrophy and were excluded. The mean age of the study cohort was 75.8 years (SD 7.9 years), with 282 (35.0%) being male (Table 1). The mean age of patients with bilateral iAMD was lower than the age of patients with nAMD in the fellow eye (*P* < 0.001, One-way ANOVA) (Table 2).

**Table 1:** Baseline summary statistics on the total cohort and by study eye diagnosis.

Characteristic	Overall, *N* = 983 eyes of 805 patients	No atrophy & no SDD, *N* = 321 eyes of 273 patients	No atrophy with SDD, *N* = 385 eyes of 323 patients	Early atrophy (iRORA) & no SDD, *N* = 114 eyes of 106 patients	Early atrophy (iRORA) with SDD, *N* = 163 eyes of 142 patients
Participant level (*N* = 805 patients)					
Age, years	75.8 (7.9)	74.0 (7.8)	77.1 (7.5)	72.8 (8.5)	78.0 (7.6)
Age, years, categories					
<75	333 (41.4%)	138 (50.5%)	114 (35.3%)	61 (57.5%)	40 (28.2%)
75–84	371 (46.1%)	116 (42.5%)	159 (49.2%)	39 (36.8%)	74 (52.1%)
85+	101 (12.5%)	19 (7.0%)	50 (15.5%)	6 (5.7%)	28 (19.7%)
Sex					
F	523 (65.0%)	168 (61.5%)	213 (65.9%)	62 (58.5%)	102 (71.8%)
M	282 (35.0%)	105 (38.5%)	110 (34.1%)	44 (41.5%)	40 (28.2%)
Bilateral eligibility	178 (22.1%)	48 (17.6%)	62 (19.2%)	8 (7.5%)	21 (14.8%)
AMD category, fellow eye					
Bilateral eligibility	178 (22.1%)	48 (17.6%)	62 (19.2%)	8 (7.5%)	21 (14.8%)
Not recorded or insufficient data	8 (1.0%)	21(7.7%)	30 (9.3%)	19 (17.9%)	16 (11.3%)
Early AMD	11 (1.4%)	5 (1.8%)	2 (0.6%)	2 (1.9%)	2 (1.4%)
Established Geographic Atrophy (cRORA)	72 (8.9%)	15 (5.5%)	18 (5.6%)	17 (16.0%)	22 (15.5%)
Neovascular AMD (nAMD)	499 (62.0%)	160 (58.6%)	202 (62.5%)	59 (55.7%)	78 (54.9%)
Healthy macula	5 (0.6%)	3 (1.1%)	2 (0.6%)	0 (0%)	0 (0%)
Other retinal disease than AMD related	32 (4.0%)	21 (7.7%)	7 (2.2%)	1 (0.9%)	3 (2.1%)
Eye level (*N* = 983 eyes)					
VA, study eye, ETDRS letters, median (IQR), mean (SD)	80.0 (75.0, 85.0), 79.8 (8.1)	81.0 (75.0, 85.0), 80.3 (7.8)	80.0 (75.0, 85.0), 79.5 (8.5)	83.0 (75.0, 85.0), 80.5 (7.4)	80.0 (75.0, 85.0), 78.9 (8.2)
Missing	33	6	18	4	5
VA, study eye, ETDRS letters, categories					
<37	3 (0.32%)	1 (0.3%)	2 (0.5%)	0 (0.0%)	0 (0.0%)
37–54	9 (0.95%)	3 (1.0%)	3 (0.8%)	1 (0.9%)	2 (1.3%)
55–69	56 (5.89%)	17 (5.4%)	25 (6.8%)	5 (4.5%)	9 (5.7%)
70–79	252 (26.5%)	73 (23.2%)	95 (25.9%)	31 (28.2%)	53 (33.5%)
80 or better	630 (66.3%)	221 (70.2%)	242 (65.9%)	73 (66.4%)	94 (59.5%)
Missing	33	6	18	4	5
VA method, study eye, %					
Missing	36 (3.66%)	6 (1.9%)	20 (5.2%)	4 (3.5%)	6 (3.7%)
Best corrected	634 (64.5%)	203 (63.2%)	273 (70.9%)	66 (57.9%)	92 (56.4%)
Glasses	305 (31.0%)	110 (34.3%)	88 (22.9%)	42 (36.8%)	65 (39.9%)
Pinhole	8 (0.81%)	2 (0.6%)	4 (1.0%)	2 (1.8%)	0 (0.0%)
Total line scans					
<49	689 (70.1%)	217 (67.6%)	281 (73.0%)	88 (77.2%)	103 (63.2%)
>=49	294 (29.9%)	104 (32.4%)	104 (27.0%)	26 (22.8%)	60 (36.8%)

*iAMD* intermediate age-related macular degeneration, *cRORA* complete retinal and retinal pigment epithelial atrophy, i*RORA* incomplete retinal and retinal pigment epithelial atrophy, *nAMD* neovascular age related macular degeneration, *SDD* subretinal drusenoid deposits, *VA* visual acuity.

**Table 2:** Comparison of VA in bilateral iAMD eyes, exudative AMD eyes and GA eyes.

	Bilateral iAMD	iAMD study eye and nAMD in non-study eye	iAMD study eye and GA in non-study eye
***N***	178	499	72
**Mean Age, years**	73.2 (8.2)	77.0 (7.6)	74.9 (8.4)
**Female, No. %**	122 (68.5%)	320 (64.1%)	24 (66.8%)
**Mean (SD) VA in fellow non-study eye**	NA	61.5 (19.4)	63.4 (21.3)
**Mean (SD) VA in study eye**	BSE: 83.5 (5.5) WSE: 79.2 (7.1)	78.8 (8.4)	80.9 (7.2)
**Paired** ***t*** **test** ***P*** **value**	Comparing better and worse eye: *p* < 0.001 Comparing left and right eye: *p* = 0.84	<0.001	<0.001
**ICC**	ICC = 0.60 for better vs worse eye. ICC = 0.55 for left vs right eye	0.05	0.10

*iAMD* intermediate age-related macular degeneration, *BSE* better seeing eye, *WSE* worse seeing eye, *GA* geographic atrophy, *ICC* intraclass correlation coefficient, *SD* standard deviation, *VA* visual acuity, *nAMD* neovascular AMD.

### AMD characteristics of the study cohort

The distribution of iAMD subtypes is shown in Table 1. Of the total eyes with iAMD, 548 (55.7%) had SDD and 277 (28.2%) had iRORA. A total of 499 (62.0%) patients had iAMD in one eye and nAMD in the other eye. 72 (8.9%) had iAMD in one eye and cRORA in the other.

In patients with bilateral iAMD, concordance in iAMD subtypes between eyes was observed in 139/178 (78.1%). In patients with nAMD in the fellow eye (*n* = 499), 339/499 (67.9%) of the study eyes with iAMD had either iRORA or SDD or both in the study eye, and iRORA was less common (137/499, 27.5%) than SDD (280/499, 56.1%) and 78 (15.6%) had co-existent iRORA and SDD.

The most frequent subtype of iAMD in eyes with nAMD in fellow eyes were those with SDD and no atrophy (202/499, 40.5%), followed very closely by eyes with no SDD and no iRORA (160/499, 32.1%). In contrast, in patients with cRORA in the fellow eye, 57/72 (79.2%) of the study eye with iAMD had either SDD or iRORA or both.

When we consider the differences in iAMD subtypes depending on the type of advanced AMD in the fellow eyes, iRORA in the study eye was more common in eyes with cRORA in the fellow eye than eyes with nAMD in the fellow eye (39/72, 54.2% vs 137/499, 27.5%; Chisq-test; *P* < 0.001).

### Profile of study cohort based on age, gender and VA

Considering eyes with iAMD only, the categories with the highest mean ages were those with SDD (Fig. S1). The mean age of those with SDD and iRORA was 78.0 years, SD 7.6 years, and SDD with no iRORA was 77.1 years, SD 7.5 years. Conversely, iRORA with no SDD (72.8 years, SD 8.5 years) and no iRORA with no SDD (74.0 years, SD 7.8 years) had lower mean ages at diagnosis. In contrast, the male: female ratio of approximately 1:2 was consistent in all subtypes of iAMD.

There were 33 eyes with missing VA data. Of the rest of the cohort (*n* = 967), the median VA of the eyes with iAMD was 80.0 (interquartile range [IQR], 75.0, 85.0) ETDRS letters (Fig. S2). Majority of the study eyes (*N* = 882 of 950, 92.8%) had a VA of at least 70 letters and 630 (66.4%) had 80 or more ETDRS letters.

**Fig. 1:**
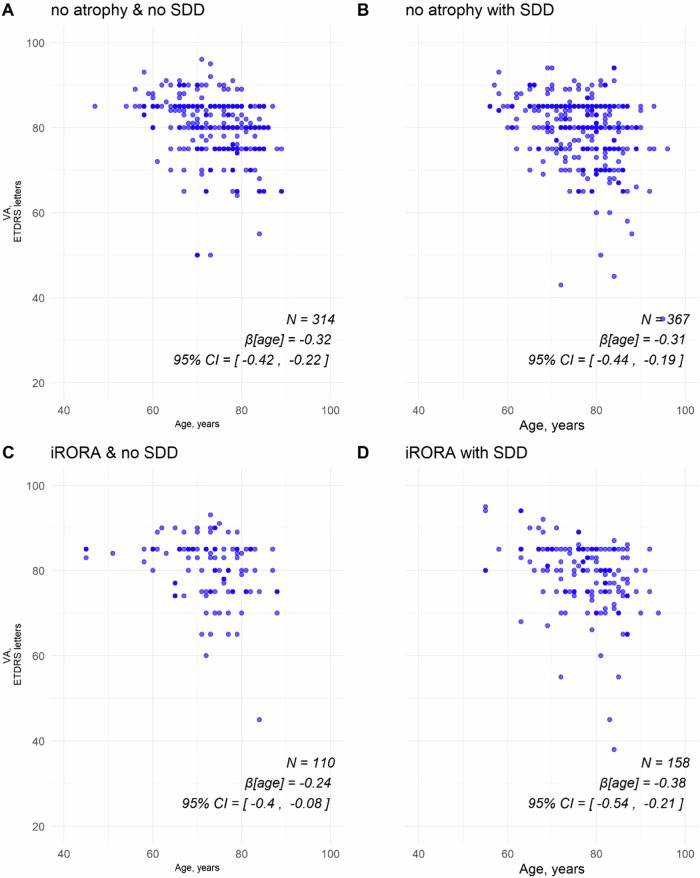
Correlation of VA and age in different iAMD subgroups. One eye with VA 20 excluded from the no atrophy & no SDD group. Beta coefficient for age obtained from linear mixed effects models, with random intercepts by participant which account for within participant correlation resulting from clustering of 2 eyes within the same participant. Participant subgroups presented in panels **A** no atrophy & no SDD, **B** no atrophy with SDD, **C** iRORA & no SDD, **D** iRORA with SDD. iRORA incomplete retinal pigment epithelium and outer retinal atrophy, SDD subretinal drusenoid deposits, VA visual acuity.

Table 2 shows that the mean VA of the eyes with bilateral iAMD was higher in the better seeing eye than the worse seeing eye (*P* < 0.001, paired *t* test). The mean VA of the study eye with iAMD of patients with nAMD in the fellow eye was 78.8 letter score (SD 8.4), and the mean VA in the study eye with iAMD of patients with GA in the fellow eye was 80.9 letters (SD 7.2). The Intraclass Correlation Coefficients (ICC) were 0.55 for comparisons between left and right eyes and 0.60 for comparisons between the better and worse eyes among bilaterally eligible eyes. The ICC of VA was 0.05 for eyes with nAMD in the fellow eye and 0.10 for eyes with GA in the fellow eye. A statistically significant negative association between age and VA was observed across all iAMD subgroups (Fig. 1). However, the interaction between age and iAMD subgroup for VA was not statistically significant, indicating a consistent association of age on VA across subgroups, as determined by the linear mixed effects model. The coefficient for the association between age and VA at baseline was β = −0.33 (95% CI −0.40, −0.26), *P* < 0.001.

### Age, sex and VA associations of presence of SDD, iRORA and by iAMD severity

Analysis of presence and absence of SDD revealed that increased age (sex-adjusted OR, 1.07 per 1 year increase in age, 95% CI 1.05–1.09; *P* < 0.001) was associated with a higher likelihood of having SDD at baseline (Table 3). Male sex (Male vs Female; unadjusted OR 0.75, 95% CI 0.56–0.99; *P* = 0.045) and increased VA (unadjusted OR 0.98 per 1 letter increase, 95% CI 0.97–0.999; *P* = 0.033) were at reduced odds of SDD presence at baseline in univariate analysis, however this did not reach statistical significance at the 5% level in adjusted analysis, though the evidence against the null hypothesis for Male sex remains moderate in adjusted analysis (adjusted OR 0.75, 95% CI 0.56–1.01; *P* = 0.057). BCVA did not reach statistical significance in univariate nor adjusted analysis for the presence of SDD at baseline. Eyes with nAMD in the fellow eye had increased odds of SDD presence in univariate analysis (unadjusted OR 1.54, 95% CI 1.05–2.29; *P* = 0.029), but this did not reach statistical significance at the 5% level after adjusting for age and sex. For the analysis on the presence of iRORA at baseline, no associations were found with age, sex, VA or BCVA (Table S2). Eyes with nAMD in the fellow eye had reduced odds of iRORA in the study eye (adjusted OR = 0.60, 95% CI 0.40–0.91; *P* = 0.015) but eyes with GA in the fellow eye had increased odds of iRORA in the study eye (adjusted OR = 3.30, 95% CI 2.00–5.48; *P* < 0.001).

**Table 3:** Univariate and age-gender adjusted analysis of SDD presence at baseline: Logistic regression via Generalised Estimating Equations (GEE) with an exchangeable correlation structure for modelling the within-participant correlation.

		Unadjusted		Adjusted^a^	
Characteristics	*N*	OR (95% CI)	*p* value	OR^a^ (95% CI)	*p* value
**Best corrected VA, study eye, per 1 letter increase**	634	0.995 (0.98–1.02)	0.63	1.003 (0.98–1.02)	0.76
**Best corrected VA, study eye, categories**	634				
*<=69*		—		—	
*70–79*		0.99 (0.60–1.64)	0.98	1.03 (0.61–1.76)	0.91
*80 or better*		0.93 (0.59–1.46)	0.75	1.05 (0.65–1.70)	0.84
**VA, study eye, per 1 letter increase**	950	0.98 (0.97–0.999)	0.033	0.99 (0.97–1.01)	0.37
**VA, study eye, categories**	950				
*<=69*		—		—	
*70–79*		0.74 (0.45–1.21)	0.22	0.92 (0.56–1.54)	0.76
*80 or better*		0.67 (0.43–1.05)	0.079	1.12 (0.61–2.07)	0.71
**Age, per 1 year increase**	983	1.07 (1.05–1.09)	<0.001	1.07 (1.05–1.09)	<0.001
**Age, years, categories**	983				
*<75*		—		—	
*75–84*		2.03 (1.48–2.78)	<0.001	1.99 (1.44–2.74)	<0.001
*85* +		4.16 (2.52–6.87)	<0.001	3.99 (2.35–6.77)	<0.001
**Sex**	983				
*F*		—		—	
*M*		0.75 (0.56–0.99)	0.045	0.75 (0.56–1.01)	0.057
**nAMD in fellow eye in eyes with unilateral eligibility**^b^	627				
*No*		—		—	
*Yes*		1.54 (1.05–2.29)	0.029	1.41 (0.94–2.11)	0.098
**GA in fellow eye in eyes with unilateral eligibility**^b^	627				
*No*		—		—	
*Yes*		1.08 (0.66–1.78)	0.77	1.16 (0.70–1.95)	0.56

*AMD* age related macular degeneration, *GA* geographic atrophy, *GEE* Generalised Estimating Equations, *OR* odds ratio, *SDD* subretinal drusenoid deposits, *VA* visual acuity, *nAMD* neovascular AMD.

^a^Adjusted for categorical age (<= 75, 75–84, 85 + ) and gender, except for age as a continuous variable where it was adjusted for gender only.

^b^Logistic regression without employing GEE were used for model fitting, as the data were based on eyes with unilateral eligibility.

Multinomial logistic regression (Table 4) analysis indicated that older age was associated with a higher likelihood of having SDD with no iRORA (OR = 1.06 [95% CI 1.03, 1.08]; *P* < 0.001) and SDD with iRORA (OR = 1.08 [95% CI 1.04, 1.11]; *P* < 0.001) relative to having no iRORA and no SDD. Additionally, females were more likely to present with iRORA and SDD compared to no iRORA and no SDD (OR = 0.63 [95% CI 0.41, 0.98]; *P* = 0.04). Eyes with iRORA with SDD had reduced VA relative to eyes with no iRORA and no SDD (OR = 0.98 [95% CI 0.96, 0.998]; *P* = 0.03). Eyes with nAMD in the fellow eye had increased odds of iAMD with no iRORA (OR = 1.95 [95% CI 1.19, 3.21]; *P* = 0.008). Eyes with GA in the fellow eye had increased odds of both iAMD with iRORA with no SDD (OR = 3.42 [95% CI 1.62,7.23]; *P* = 0.001) and iAMD with iRORA with SDD (OR = 3.41 [95% CI 1.69, 6.90]; *P* < 0.001). The analysis remained similar for the association with BCVA but the *p* value did not reach statistical significance.

**Table 4:** Unadjusted multinomial logistic regression analysis via Generalised Estimating Equations (GEE) with time exchangeable correlation structure to model iAMD severity group at baseline: Local Odds Ratio of demographic and baseline vision characteristics by iAMD severity group, with iAMD with no iRORA and no SDD as the reference category.

Characteristic	iAMD with no iRORA with SDD, *N* = 385		iAMD with iRORA with no SDD, *N* = 114		iAMD with iRORA with SDD, *N* = 163	
	Local OR (95% CI)	*P*-value	Local OR (95% CI)	*P*-value	Local OR (95% CI)	*P*-value
**Age, per 1 year increase**	1.06 (1.03, 1.08)	<0.001	0.98 (0.96, 1.01)	0.22	1.08 (1.04, 1.11)	<0.001
**Age, years, categories**						
*<75*	Ref	**—**	Ref	**—**	Ref	**—**
*75–84*	1.72 (1.21, 2.45)	0.002	0.77 (0.48, 1.23)	0.27	2.32 (1.45, 3.69)	<0.001
*85* +	3.26 (1.82, 5.86)	<0.001	0.74 (0.29, 1.90)	0.53	5.41 (2.72, 10.74)	<0.001
**Gender**						
*Female*	Ref	**—**	Ref	**—**	Ref	**—**
*Male*	0.85 (0.61, 1.19)	0.35	1.19 (0.76, 1.87)	0.45	0.63 (0.41 0.98)	0.042
**VA, per 1 letter increase**	0.99 (0.97, 1.004)	0.13	1.004 (0.97, 1.04)	0.79	0.98 (0.96, 0.998)	0.030
**VA, ETDRS letter, categories**						
*<=69*	Ref	**—**	Ref	—	Ref	—
*70–79*	0.75 (0.42, 1.32)	0.32	1.42 (0.51, 3.96)	0.51	0.91 (0.49, 1.69)	0.77
*80 or better*	0.75 (0.42, 1.32)	0.18	1.13 (0.43, 3.01)	0.81	0.68 (0.38, 1.25)	0.21
**Best corrected VA, per 1 letter increase**	0.99 (0.97, 1.02)	0.59	0.98 (0.95, 1.02)	0.35	0.97 (0.95, 1.01)	0.11
**Best corrected VA, category**						
*<=69*	Ref	—	Ref	—	Ref	—
*70–79*	1.06 (0.54, 2.09)	0.87	0.89 (0.28, 2.88)	0.85	0.76 (0.33, 1.79)	0.54
*80 or better*	0.93 (0.50, 1.73)	0.82	0.54 (0.18, 1.64)	0.28	0.52 (0.23, 1.16)	0.11
**nAMD in fellow eye in eyes with unilateral eligibility**^a^						
*No*	Ref	—	Ref	—	Ref	—
*Yes*	1.95(1.19, 3.21)	0.008	0.80 (0.45,1.45)	0.47	0.87 (0.50,1.49)	0.61
**GA in fellow eyes in eyes with unilateral eligibility**^a^						
*No*	Ref	—	Ref	—	Ref	—
*Yes*	1.08 (0.53, 2.20)	0.84	3.42 (1.62,7.23)	0.001	3.41 (1.69, 6.90)	<0.001

The reference category for the multinomial logistic regression model was iAMD with no iRORA and no SDD (*N* = 321).

*iAMD* intermediate age related macular degeneration, *GA* geographic atrophy, *GEE* Generalised estimating Equations, *iRORA* incomplete retinal and retinal pigment epithelial atrophy, *VA* visual acuity, *nAMD* neovascular AMD.

^a^Multinomial logistic regression models without employing GEE were used to fit the data as analysis was based on eyes with unilateral eligibility and therefore can be interpreted as odds ratios.

## Discussion

This study reveals a few points that are relevant for future trial designs on intervention for iAMD. Firstly, those with SDD were older compared to those with bilateral iAMD (mean 73.2 years), iAMD in only the study eye and fellow eye having nAMD (77.0 years) or GA (74.9 years). The phenotypes with iRORA were not influenced by age. These findings suggest that if trial cohorts are skewed to the older age-groups (75 years or above), a high proportion are likely to have SDD. Similarly, the mean ages between the sham and intervention arm need to be comparable too. The mean age of the sham groups of both the risuteganib (78.8 in sham vs 75.9) in risuteganib and the LIGHTSITE III trials (77.1 in sham vs 74.4 in photobiomodulation) were 3 years older than the intervention arms [10, 19].

Although these differences are numerically small, eyes with SDD have a higher risk of progression to GA [14] and it remains unclear whether the visual function in these eyes can be reversed. In the subthreshold nanosecond laser intervention in iAMD (LEAD trial), the median BCVA was 83 letters (20/25) and 24% had SDD. Eyes with SDD showed higher progression rate to advanced AMD compared to those without SDD suggesting that those with SDD may have different aetiopathogensis compared to those without SDD [9].

Secondly, eyes with iRORA are three times more likely to have GA in the fellow eye and so enriching study cohort with iRORA patients is likely to have more event rates if the endpoint is defined as transition to GA or cRORA. In addition, iAMD eyes with fellow eye having nAMD are less likely to have iRORA. The concordance in subtypes of iAMD in eyes with bilateral iAMD was 78.1% irrespective of age.

Third, the median VA of our study cohort with iAMD was 80.0 (75.0, 85.0) ETDRS letters. When the iAMD eyes was classified based on the condition of the fellow eye into iAMD/iAMD; iAMD/nAMD and iAMD/GA, we found that there was significant difference in mean VA comparing better and worse-seeing eyes in bilateral iAMD in these categories. These findings are in keeping with an EMR report on 83,425 patients from UK clinical services [8].

Fourth, including the worse seeing eye of bilateral iAMD group in prevention trials may result in a more homogeneous trial cohort as the VA of worse seeing eye is more likely to be closer to the iAMD in fellow eyes with advanced AMD.

Next, only 7.2% of our cohort with iAMD had a VA of <70 letters. Trial designs need to consider whether the aim is to assess 15 letter gain or loss. The baseline VA in most iAMD eyes is high and so an outcome of preventing 15 letter loss is more appropriate. For example, in the AREDS trial, participants with either bilateral large drusen or late AMD in one eye and large drusen in the fellow eye were enroled and the baseline mean BCVA was 84.5 letters. Therapy with the AREDS formulation reduced the risk of at least a 15-letter loss by 19% at 5 years (estimated probability of progression was 29% for placebo versus 23% for intervention) [20]. Similarly, at baseline, in the AREDS 2 trial cohort with bilateral iAMD, 87.6% had Snellen 20/40 or better and 36.8% were Snellen 20/20 or better [21].

In contrast, the mean baseline BCVA in the risuteganib trial of 67.1 and 64.4 letters and the Low-Level Light Therapy trial by Borrelli et al. of 66.2 and 64 letters in the sham and intervention groups, respectively, where the outcome is a mean change in VA from the low baseline VA [19, 22]. In the risuteganib trial, 20% gained >15 letters at week 28, which is a regulatory end-point [19]. Post-hoc analysis showed that greater outer retinal and photoreceptor thickness and volume and smaller ellipsoid zone defect area in the central 1 mm zone at baseline were associated with increased BCVA response to risuteganib [6]. Therefore, using these structural markers that determine VA as screening tools may help identify the subgroup of iAMD that is likely to benefit from a gain in VA despite a low baseline VA.

Our study has several strengths. Data were drawn from a large cohort of patients across Europe with imaging done on Spectralis. Statistical adjustments were applied to account for the analysis of both eyes from the same participant. The results also mirror some of the large studies on iAMD.

However, our study also has several limitations. The sample is obtained from retinal clinics in hospitals and are therefore biased towards those undergoing treatment for nAMD. There were minimal data on other risk factors other than age, gender and disease status of the fellow eye [23]. Other ocular imaging prognostic markers were also not individually graded in this study [24, 25]. Furthermore, VA could have been influenced by other factors not collected (i.e. presence of cataracts) and the grading of iAMD subclassifications were performed at the site level and while efforts were made to ensure consistency, a centralized reading centre was not utilised, which may introduce variability in classification. Additionally, the use of AREDS supplement was not recorded [26]. These factors should be considered in the interpretation of the findings and in the design of future studies to ensure a more comprehensive risk factor analysis.

In conclusion, baseline age, presence of SDD and/or iRORA, better or worse seeing eye in bilateral iAMD, and fellow eye status in unilateral iAMD need to be considered in future clinical trials evaluating preventive or treatment options for iAMD.

## Summary

### What was known before

- Various phenotypes of intermediate AMD exist.
- Eyes with iAMD with subretinal drusenoid deposits (SDD) and incomplete retinal and retinal pigment epithelial atrophy (iRORA) have a high risk of progression to advanced AMD.
- Clinical trials evaluating interventions in iAMD often include either one eye of bilateral iAMD, which may be the better or worse seeing eye, or the second eye with iAMD in patients where unilateral advanced AMD. These may have consequences in final VA outcome.

### What this study adds

- We further classified eyes with iAMD into four groups based on presence of iRORA and SDD on multimodal imaging. Eyes with SDD and iRORA have worse visual acuity compared to those with no SDD or iRORA.
- When the iAMD eyes was classified based on the condition of the fellow eye into iAMD/iAMD; iAMD/eAMD and iAMD/GA, we found that there was significant difference in mean VA comparing better and worse-seeing eyes in bilateral iAMD in these categories.
- We also observed that only 7.2% of our cohort with iAMD had a visual acuity of <70 letters.
- Baseline age, presence of SDD and/or iRORA, better or worse seeing eye in bilateral iAMD, and fellow eye status in unilateral iAMD need to be considered in future clinical trials evaluating preventive or treatment options for iAMD.

## Supplementary information

Table S1. Proportion of patients with different combinations of AMD severity in both eyes

Table S2. Unadjusted and adjusted analysis for presence of iRORA at baseline: Logistic regression via Generalised Estimating Equations (GEE) with an exchangeable correlation structure for modelling wi

Figure S1. Age by iAMD category

Figure S2. Visual Acuity by iAMD category

## Data Availability

The data that support the findings of this study are not openly available due to reasons of sensitivity and are available from EVICR.net upon reasonable request.
