# Supplementary material for: Visual acuity in various phenotypes of intermediate age related macular degeneration (AMD) in a multicentre cohort study in Europe- INTERCEPT-AMD report 1
Source: Eye (Lond). 2025 Jul 19;39(14):2655–63. doi: 10.1038/s41433-025-03895-y (PMC12446473; doi:10.1038/s41433-025-03895-y)
Supplement: Supplementary file 1 — Table S1. Proportion of patients with different combinations of AMD severity in both eyes [file 41433_2025_3895_MOESM1_ESM.docx]

**Table S1. Proportion of patients with different combinations of AMD severity in both eyes**

| Condition | No atrophy and no SDD | No atrophy with SDD | iRORA with no SDD | iRORA with SDD | nAMD | cRORA | early AMD | Other retinal disease than AMD related | healthy macula | insufficient data or missing |
| --- | --- | --- | --- | --- | --- | --- | --- | --- | --- | --- |
| No atrophy and no SDD | 48(5.9%) | 6(0.7%) | 11(1.3%) | 0(0.0%) | 160(19.5%) | 15(1.8%) | 5(0.6%) | 21(2.6%) | 3(0.4%) | 4(0.5%) |
| No atrophy with SDD |  | 62(7.6%) | 6(0.7%) | 16(2.0%) | 202(24.7%) | 18(2.2%) | 2(0.2%) | 7(0.9%) | 2(0.2%) | 2(0.2%) |
| iRORA with no SDD |  |  | 8(1.0%) | 0(0.0%) | 59(7.2%) | 17(2.1%) | 2(0.2%) | 1(0.1%) | 0(0.0%) | 2(0.2%) |
| iRORA with SDD |  |  |  | 21(2.6%) | 78(9.5%) | 22(2.7%) | 2(0.2%) | 3(0.4%) | 0(0.0%) | 0(0.0%) |
| nAMD |  |  |  |  | 0(0.0%) | 0(0.0%) | 0(0.0%) | 0(0.0%) | 0(0.0%) | 0(0.0%) |
| cRORA |  |  |  |  |  | 0(0.0%) | 0(0.0%) | 0(0.0%) | 0(0.0%) | 0(0.0%) |
| early AMD |  |  |  |  |  |  | 0(0.0%) | 0(0.0%) | 0(0.0%) | 0(0.0%) |
| Other retinal disease than AMD related |  |  |  |  |  |  |  | 0(0.0%) | 0(0.0%) | 0(0.0%) |
| healthy macula |  |  |  |  |  |  |  |  | 0(0.0%) | 0(0.0%) |
| insufficient data or missing |  |  |  |  |  |  |  |  |  | 0(0.0%) |

**Abbreviations;** SDD-sub retinal drusenoid deposit ; iRORA- incomplete retinal and retinal pigment epithelial atrophy; ; AMD-age related macular deneration; cRORA - complete retinal and retinal pigment epithelial atrophy; nAMD- neovascular AMD
