# Supplementary material for: Visual acuity in various phenotypes of intermediate age related macular degeneration (AMD) in a multicentre cohort study in Europe- INTERCEPT-AMD report 1
Source: Eye (Lond). 2025 Jul 19;39(14):2655–63. doi: 10.1038/s41433-025-03895-y (PMC12446473; doi:10.1038/s41433-025-03895-y)
Supplement: Supplementary file 2 — Table S2. Unadjusted and adjusted analysis for presence of iRORA at baseline: Logistic regression via Generalised Estimating Equations (GEE) with an exchangeable correlation structure for modelling wi [file 41433_2025_3895_MOESM2_ESM.docx]

**Table S2. Unadjusted and adjusted analysis for presence of iRORA at baseline: Logistic regression via Generalised Estimating Equations (GEE) with an exchangeable correlation structure for modelling within-participant correlation structure**

|  |  | **Unadjusted** | | **Adjusted*** | |
| --- | --- | --- | --- | --- | --- |
| **Characteristic** | **N** | **OR (95% CI)** | **p-value** | **OR (95% CI)** | **p-value** |
| **Best corrected VA, study eye, per 1 letter increase** | 634 | 0.98 (0.96 - 1.01) | 0.13 | 0.98 (0.96 - 1.01) | 0.13 |
| **Best corrected VA, study eye, categories** | 634 |  |  |  |  |
| *<=69* |  | — |  | — |  |
| *70-79* |  | 0.80 (0.39 - 1.66) | 0.56 | 0.82 (0.39 - 1.71) | 0.59 |
| *80 or better* |  | 0.55 (0.28 - 1.10) | 0.093 | 0.55 (0.27 - 1.12) | 0.10 |
| **VA, study eye, per 1 letter increase** | 950 | 0.995 (0.98 - 1.01) | 0.55 | 0.996 (0.98 - 1.01) | 0.70 |
| **VA, study eye, categories** | 950 |  |  |  |  |
| *<=69* |  | — |  | — |  |
| *70-79* |  | 1.42 (0.82 - 2.47) | 0.21 | 1.45 (0.83 - 2.55) | 0.19 |
| *80 or better* |  | 1.05 (0.62 - 1.79) | 0.85 | 1.10 (0.63 - 1.91) | 0.74 |
| **Age, per 1 year increase** | 983 | 1.002 (0.98 - 1.02) | 0.81 | 1.003 (0.98 -1.02) | 0.79 |
| **Age, years, categories** | 983 |  |  |  |  |
| *<75* |  | — |  | — |  |
| *75-84* |  | 1.04 (0.76 - 1.42) | 0.83 | 1.04 (0.76 - 1.42) | 0.82 |
| *85+* |  | 1.28 (0.80 - 2.05) | 0.30 | 1.29 (0.80 - 2.06) | 0.30 |
| **Sex** | 983 |  |  |  |  |
| *F* |  | — |  | — |  |
| *M* |  | 0.90 (0.66 - 1.23) | 0.50 | 0.90 (0.66 - 1.22) | 0.49 |
| **nAMD in fellow eye in eyes with unilateral eligibility**** | 627 |  |  |  |  |
| *No* |  | — |  | — |  |
| *Yes* |  | 0.61 (0.41 - 0.92) | **0.017** | 0.60 (0.40 - 0.91) | **0.015** |
| **GA in fellow eye in eyes with unilateral eligibility**** | 627 |  |  |  |  |
| *No* |  | — |  | — |  |
| *Yes* |  | 3.28 (1.99 - 5.44) | **<0.001** | 3.30 (2.00 - 5.48) | **<0.001** |

*Adjusted for categorical age and gender, except for the variable age as a continuous variable where it was adjusted for gender only

** Logistic regression without employing GEE were used for model fitting, as the data were based on eyes with unilateral eligibility.

Abbreviations: AMD age related macular degeneration; GA-geographic atrophy; GEE-Generalised estimating Equations; iRORA- incomplete retinal and retinal pigment epithelial atrophy; OR-Odds Ratio; VA-visual acuity; nAMD- neovascular AMD.
