# Supplementary figures and images for: Visual acuity in various phenotypes of intermediate age related macular degeneration (AMD) in a multicentre cohort study in Europe- INTERCEPT-AMD report 1
Source: Eye (Lond). 2025 Jul 19;39(14):2655–63. doi: 10.1038/s41433-025-03895-y (PMC12446473; doi:10.1038/s41433-025-03895-y)

**Figure S1. Age by iAMD category**


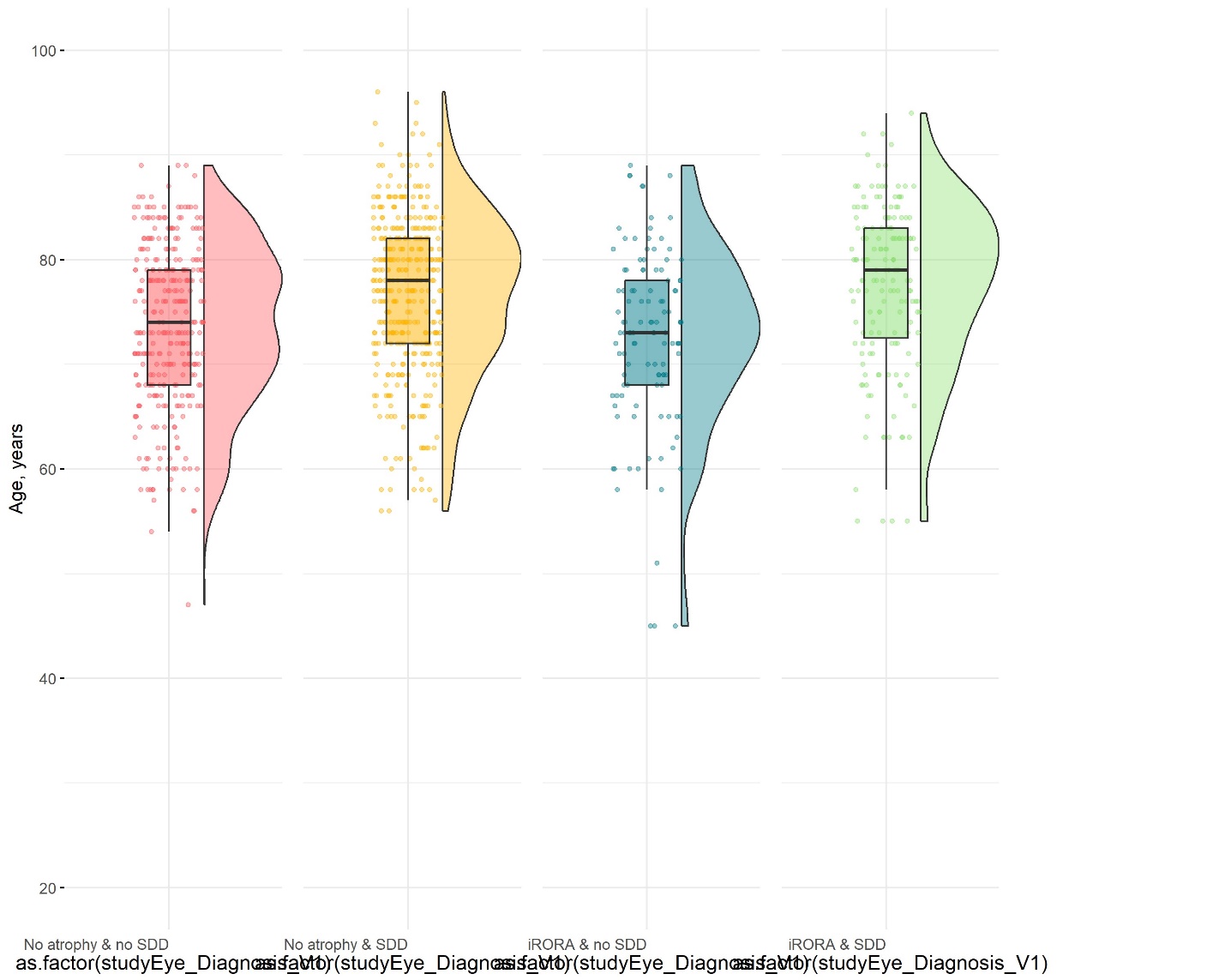

Supplement: Supplementary file 3 — Figure S1. Age by iAMD category [file 41433_2025_3895_MOESM3_ESM.docx]

**Figure S2. Visual Acuity by iAMD category**


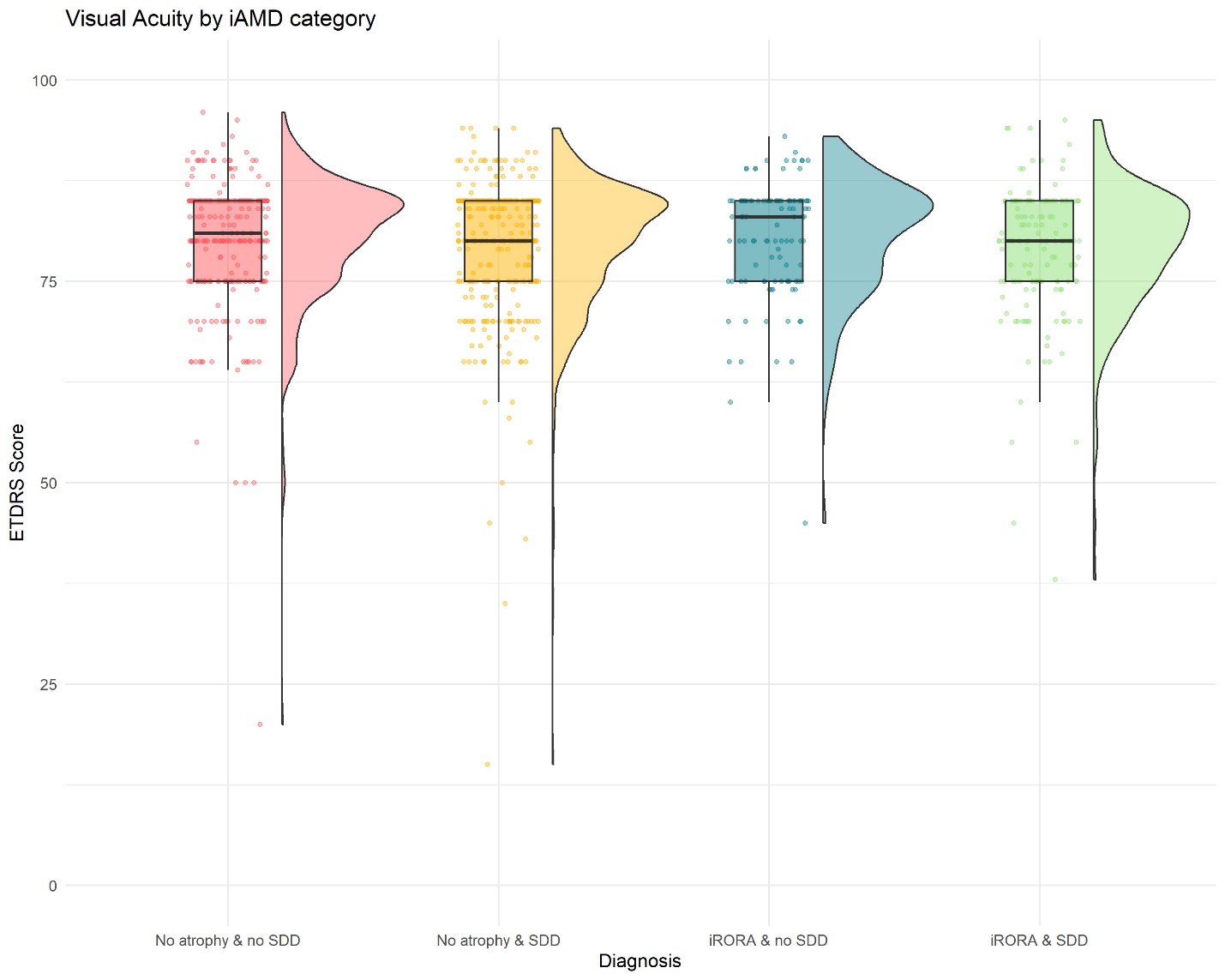

Supplement: Supplementary file 4 — Figure S2. Visual Acuity by iAMD category [file 41433_2025_3895_MOESM4_ESM.docx]
